# Overexpression of *PavbHLH28* from *Prunus avium* enhances tolerance to cold stress in transgenic *Arabidopsis*

**DOI:** 10.1186/s12870-023-04666-1

**Published:** 2023-12-18

**Authors:** Xuejiao Cao, Zhuang Wen, Tianjiao Shen, Xiaowei Cai, Qiandong Hou, Chunqiong Shang, Guang Qiao

**Affiliations:** 1 Key Laboratory of Plant Resource Conservation and Germplasm Innovation in Mountainous Region (Ministry of Education), College of Life Sciences/Institute of Agro-bioengineering, Guizhou University, Guiyang, Guizhou Province 550025 China; 2 College of Forestry, Guizhou University, Guiyang, Guizhou Province 550025 China

**Keywords:** bHLH transcription factor, Sweet cherry, Cold stress, Antioxidant enzymes, ROS

## Abstract

**Background:**

The basic helix-loop-helix (*bHLH*) gene family is one of plants’ largest transcription factor families. It plays an important role in regulating plant growth and abiotic stress response.

**Results:**

In this study, we determined that the *PavbHLH28* gene participated in cold resistance. The *PavbHLH28* gene was located in the nucleus and could be induced by low temperature. Under the treatment of ABA, PEG, and GA_3_, the transcript level of *PavbHLH28* was affected. At low temperature, overexpression of the *PavbHLH28* gene enhanced the cold resistance of plants with higher proline content, lower electrolyte leakage (EL) and malondialdehyde (MDA) content. Compared with the WT plants, the transgenic plants accumulated fewer reactive oxygen species (ROS), and the activity and expression levels of antioxidant enzymes were significantly increased. The expression of proline synthesis enzyme genes was up-regulated, and the transcripts levels of degradation genes were significantly down-regulated. The transcripts abundance of the cold stressed-related genes in the C-repeat binding factor (CBF) pathway was not significantly different between WT plants and transgenic plants after cold stress. Moreover, the PavbHLH28 could directly bind to the *POD2* gene promoter and promote its gene expression.

**Conclusions:**

Overall, *PavbHLH28* enhanced the cold resistance of transgenic plants through a CBF-independent pathway, which may be partly related to ROS scavenging.

**Supplementary Information:**

The online version contains supplementary material available at 10.1186/s12870-023-04666-1.

## Introduction

 Many extreme environments can affect plant growth and development, such as high and low temperature, drought, high salinity, etc. To survive, plants have evolved a series of regulatory mechanisms to adapt to environmental changes [1, 2]. Throughout its life cycle, plants are affected by environmental temperature. In addition to regulating plant growth, temperature also affects its geographical distribution and yield [3]. There are two types of cold stress: low-temperature stress (0–15 °C) and freezing stress (< 0 °C), and plant stress resistance changes with species differences and temperature changes [4]. Low temperature can cause functional damage and dysfunction of cells, mainly affecting plant respiration and photosynthesis while f reezing generally freezes cells and damages cell membranes, finally causing plant death [5, 6].

It was previously widely accepted that temperature affects cell membrane fluidity, changes in the cytoskeleton, and calcium entry, with a series of downstream reactions [7, 8]. The three major cold-responsive genes in plants are *Inducer of CBF Expression* (*ICE*), *C-repeat binding factors* (*CBF*), and *Cold-Regulated genes* (*CORs*) [9]. In most plants, these three named key players mimic an essential signaling pathway, the *ICE*-*CBF*-*COR* cascade, a cold-responsive pathway that relieves cold stress in plants [10, 11]. The *CBF*s can be rapidly and highly induced by low-temperature [3]. The *CBF* genes directly combine with the promoter of the *CORs* and induces their expression, thereby improving cold resistance [12, 13].

At the same time, the *CBF* gene is also regulated by upstream transcription factors. *ICE1* is the first identified *CBF* transcriptional activator and belongs to the MYC subfamily of the *bHLH* gene family [4]. Presently, *ICE1* is one of the best transcriptional activators of *CBF* genes [14]. *ICE1* can bind to the promoter of *CBF1-3* to promote their expression under cold stress [15]. The *ice1* mutant showed impaired freezing tolerance and significant defects induced by *CBF* genes, whereas overexpression of *ICE1* promotes upregulated expression of *CBF1-3* [16, 17]. Many reports have shown that *bHLH* can participate in the regulation of low-temperature stress. For example, in *Arabidopsis*, *ICE1*, which belongs to the *bHLH* transcription factor family, can activate *CBF3* and *COR* genes in response to low-temperature [18]. The apple MdCIbHLH1 protein binds to the *MdCBF2* promoter and up-regulates *MdCBF2* expression dependent on the CBF pathway to improve cold tolerance in transgenic apple plants [19]. In *Prunus Mume* and rice, cold stress specifically induced the expression of genes such as *PmbHLH57* and *OsbHLH1* [20, 21]. In addition, in sweet cherry and peach, the *bHLH* gene is involved in anthocyanin synthesis [22, 23].

Cold stress will produce more substances harmful to cells, one of them is the accumulation of ROS. ROS is a toxic molecule that causes oxidative damage to proteins, lipids and DNA [24]. On the other hand, the increase of ROS during stress is also considered as a signal to activate the stress response pathway [25]. Therefore, the lower ROS level of plants after low-temperature stress can enhance the cold resistance of plants. When subjected to cold stress, plants will maintain low levels of ROS by regulating antioxidant enzymes SOD, POD and CAT to protect plants from oxidative damage [26]. The bHLH proteins family is one of the most important transcription factor families, involved in a variety of metabolic and developmental processes [27]. In addition to *ICE* participating in the cold signal transduction pathway, some bHLH proteins can also improve the resistance of plants to cold stress. Overexpression of rice *OrbHLH001* enhances cold resistance in transgeni*c Arabidopsis.* But unlike the function of ICE, *OrbHLH001* is a *CBF*-independent cold response pathway [28]. In citrus, studies have found that *CsbHLH18* can directly regulate the antioxidant gene *CsPOD* to regulate ROS homeostasis, thereby playing an active role in cold tolerance [29]. Previous studies had found that bHLHs, as positive regulators, participate in various ways in which plants adapt to stress [30, 31]. Overexpression of *AtbHLH112* significantly enhances ROS scavenging ability to reduce the accumulation of ROS in guard cells under ABA, salt and osmotic stress conditions [27]. In Tamarisk, *ThbHLH1* is highly expressed under high salt induction, and significantly increases POD and SOD activities [27]. Additional study found that *GhbHLH18* can activate the expression of the peroxidase gene *GhPER8* to improve the stress resistance of cotton [32]. Consequently, a key indicator to assess a plant’s capacity to withstand stress is by evaluating the ability to eliminate ROS. Revealing the regulation of bHLH transcription factors in the ROS clearance mechanism can expand the understanding of bHLH functions.

The sweet cherry (*Prunus avium* L.), a fruit tree that is widely grown for its economic value worldwide, is susceptible to adverse climates such early spring low temperatures and late frosts. This can lead to low yields and instability, which can have an enormous adverse effect on the tree’s economic benefits [17]. In the previous study, we conducted genome-wide identification and expression analysis of the *bHLH* genes of sweet cherry, and identified *PavbHLH28* responses to low temperature [33]. The current study proceeded to investigate the role of *PavbHLH28*. Our results showed that *PavbHLH28* could actively respond to low temperature and enhance the cold tolerance of transgenic *Arabidopsis*. Moreover, the PavbHLH28 could directly bind to the promoter of *PavPOD2* and enhance its gene expression. Overall, rather than a CBF-dependent cold stress response mechanism, *PavbHLH28* improved cold resistance in sweet cherries. This was attributed, at least in part, to the influence of the dynamic balance of ROS through antioxidant enzyme gene regulation. Our findings provided important clues for elucidating the function of *PavbHLHs* and laid the groundwork for investigating genes that were important for genetic manipulation.

## Result

### Cloning and characterization analysis of *PavbHLH28*

**Fig. 1:**
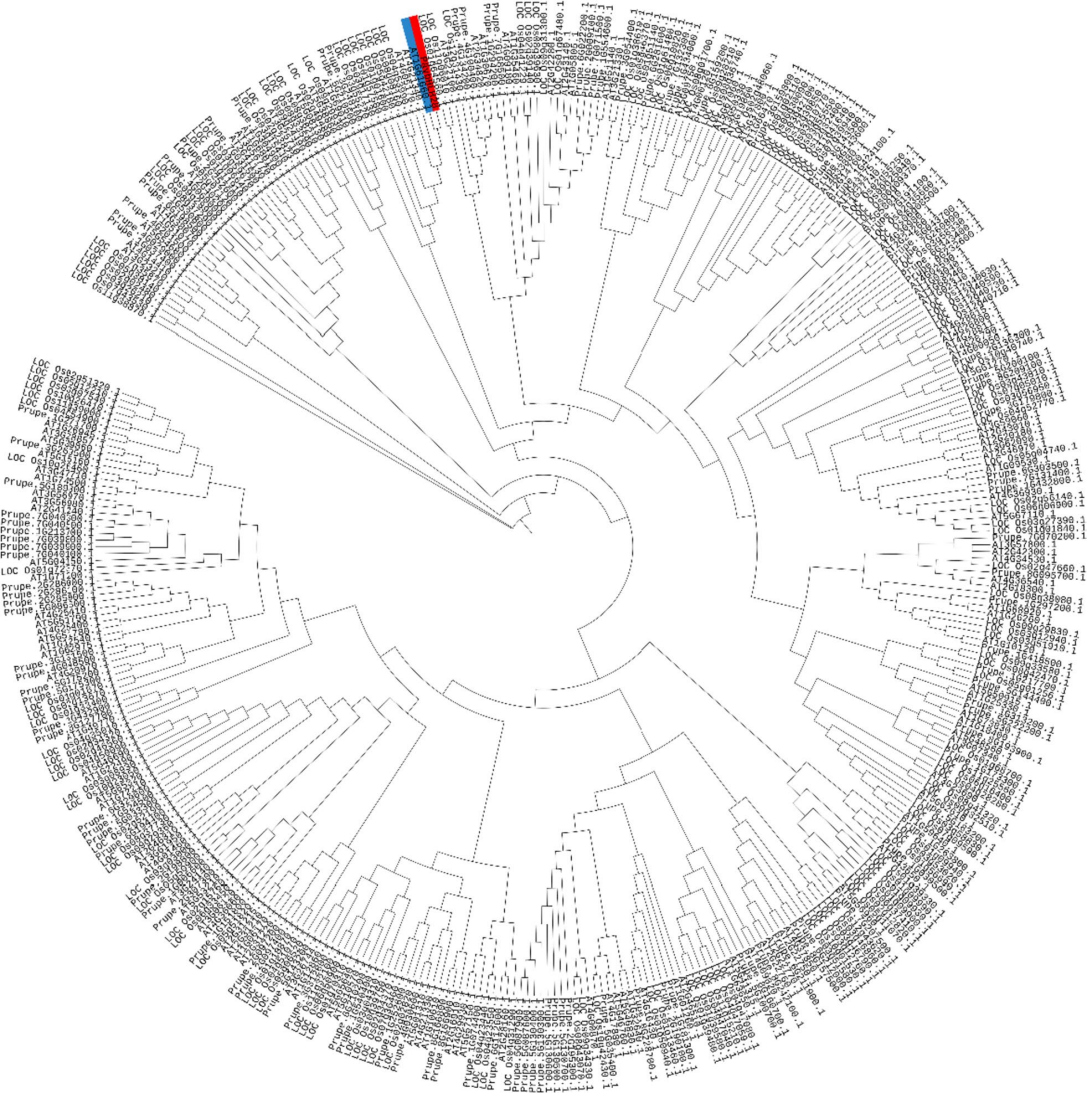
Phylogenetic tree analysis of *PavbHLH28* with *Arabidopsis thaliana*, *Oryza sativa* and *Prunus persica*

**Fig. 2:**
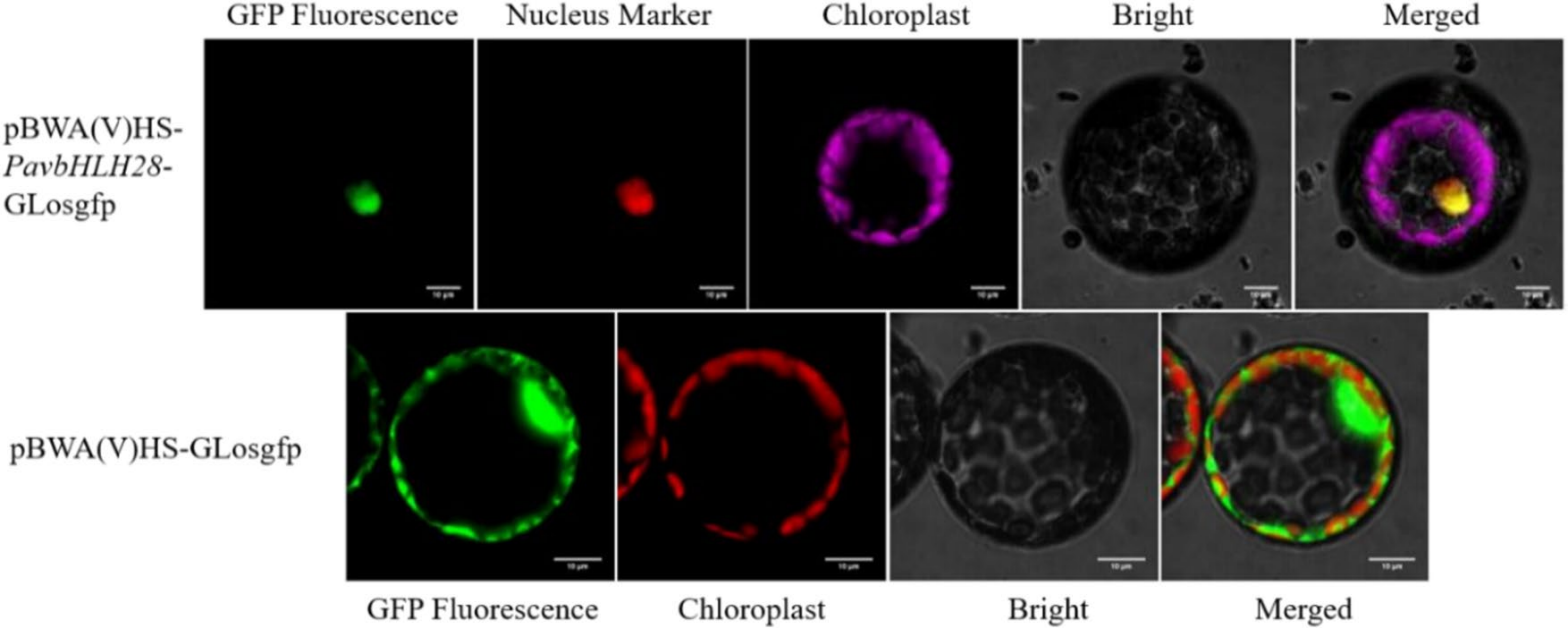
Subcellular localization of PavbHLH28 protein. pBWA(V)HS-GLosgfp and pBWA(V)HS-*PavbHLH28*-GLosgfp represent empty vector and *PavbHLH28* vector, respectively. GFP Fluorescence represents GFP fluorescence signal. Nucleus marker represents nuclear localization signal fluorescence. Chloroplast represents chloroplast fluorescence signal, the fluorescence result of *PavbHLH28* vector uses pseudo color purple to distinguish nuclear localization signal

The open reading frame (ORF) length of *PavbHLH28* was 1308 bp, which encoded 435 amino acids, the molecular weight of 46.51 kDa and an isoelectric point of 6.30. The evolutionary tree analysis showed that all branches contained bHLH genes from different species with no significant correlation with species. The *PavbHLH28* gene has the highest homology to the *AtbHLH112* (*AT1G61660*) (Fig. 1). Using *Arabidopsis* protoplast cells, a transient expression assay was used to determine the location of the gene’s function within the cell. As shown in Fig. 2, the GFP fluorescence of pBWA(V)HS-GLosgfp was displayed in the whole cell. The fluorescence location of pBWA(V)HS-*PavbHLH28*-GLosgfp completely overlapped with the nuclear localization marker fluorescence, indicating that the PavbHLH28 protein was localized in the nucleus (Fig. 2).

### Expression profiles of *PavbHLH28* in sweet cherry

**Fig. 3:**
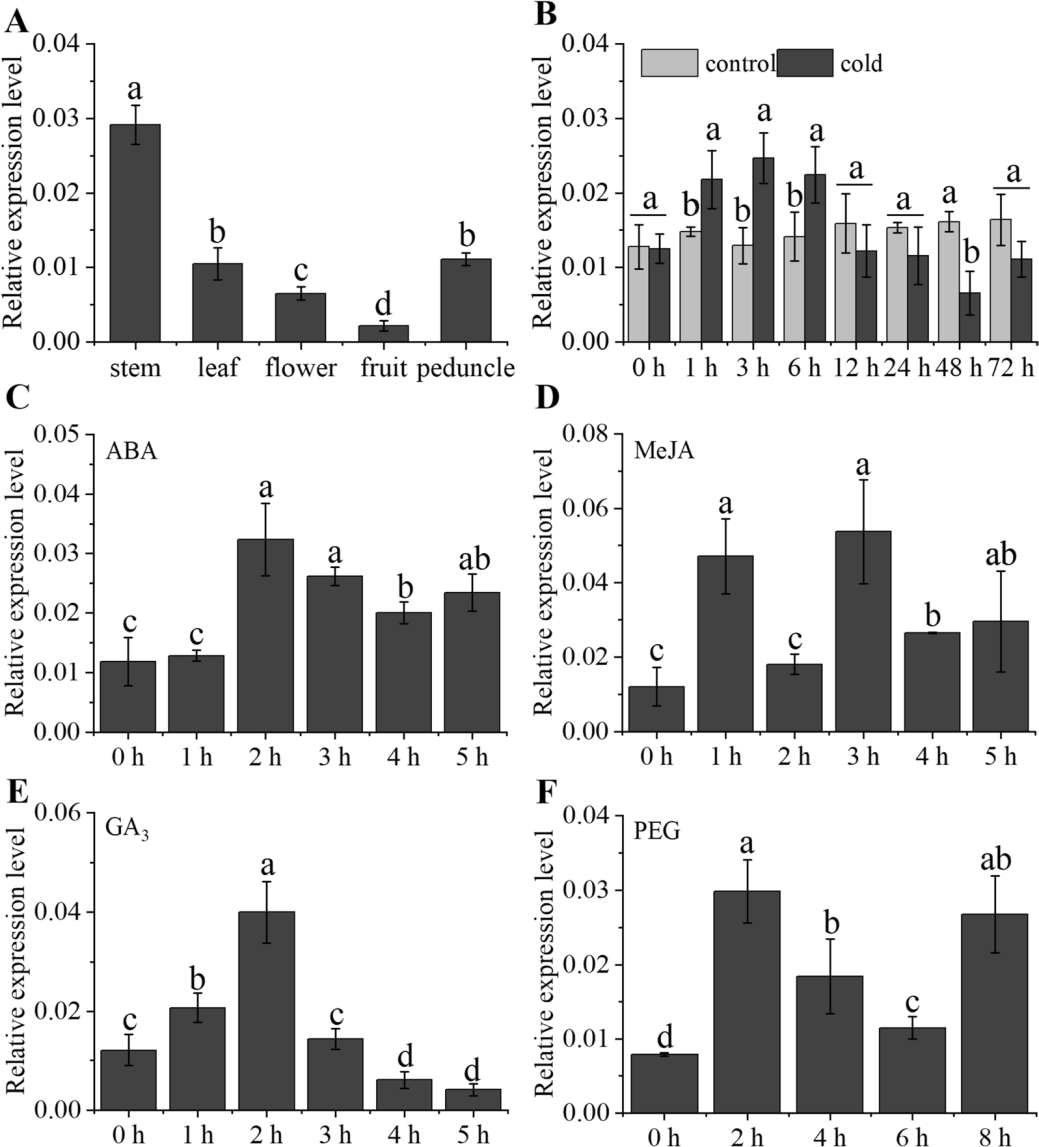
The qRT-PCR analysis of *PavbHLH28* expression patterns in sweet cherry. **A** represents *PavbHLH28* gene expression analysis in different tissues, including stem, leaf, flower, fruit and peduncle. **B**, **C**, **D**, **E**, **F** represent the gene expression analysis of *PavbHLH28* under different treatments, including cold, ABA, MeJA, GA_3_ and PEG treatments

To reveal gene expression profiles in different tissues, stem, leaf, flower, fruit and fruit stalk were tested with qRT-PCR. The results showed that the highest expression was detected in the stem and the least accumulation in the fruit. Moreover, the expression levels of *PavbHLH28* under phytohormone treatment were analyzed (Fig. 3A). Exposure to GA_3_ and ABA treatments, the *PavbHLH28* reached a peak after 2 h treatment, and then gradually decreased (Fig. 3C, E). After MeJA treatment, the expression of *PavbHLH28* gene increased for 1 h, then the gene expression abundance decreased, and the gene expression increased again after 3 h (Fig. 3D). For cold stress, the gene expression increased significantly after 1 h treatment, reached a peak at 3 h, and gradually decreased after 12 h (Fig. 3B). Under the drought stress induced by PEG, the *PavbHLH28* expression reached the maximum after 2 h treatment, then decreased, and increased again after 8 h, and both were significantly higher than the control (Fig. 3F).

### The expression analysis and cold tolerance analysis of *PavbHLH28* overexpressed transgenic plants

**Fig. 4:**
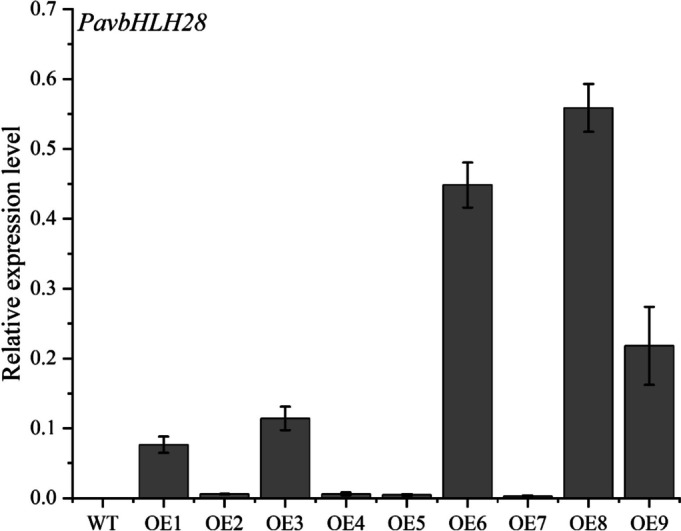
The *PavbHLH28* expression analysis of transgenic line. The OE1-9 represents nine *PavbHLH28* transgenic lines, respectively

**Fig. 5:**
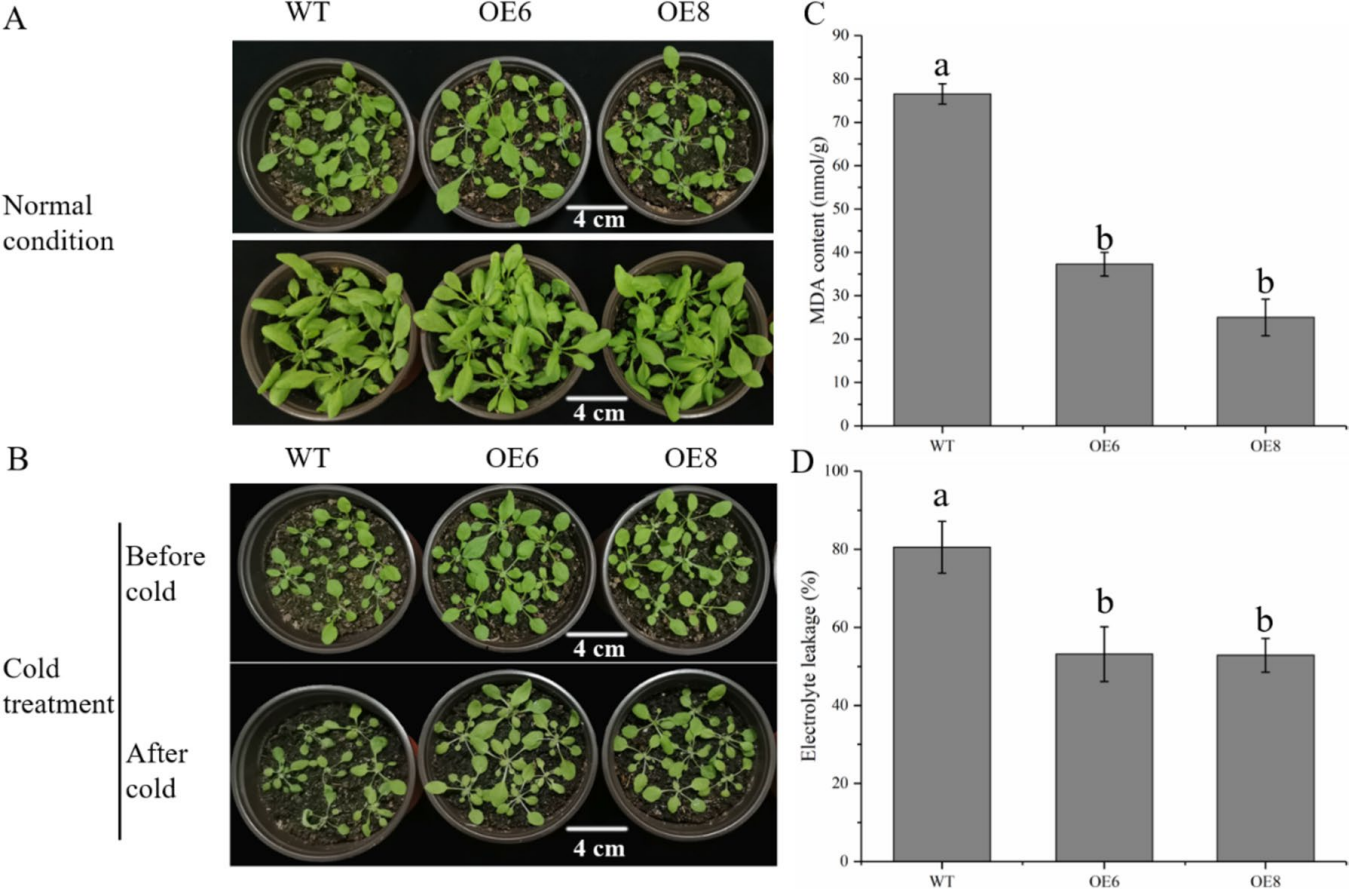
Overexpression of *PavbHLH28* enhanced cold tolerance in transgenic *Arabidopsis*. **A** and **B** represent the growth of transgenic strains and WT plants under normal conditions and cold treatment, respectively. **C** and **D** represent MDA content and electrolyte leakage of transgenic and WT plants after cold treatment, respectively. Different letters indicate the significant differences of three replicates as determined by SPSS software (*p* < 0.05)

To ascertain the transgenic strains’ expression level, we evaluated the *PavbHLH28* gene abundance in each transgenic strain; OE8 and OE6 had the highest expression abundances (Fig. 4). For instance, OE6 and OE8 were chosen for further investigation. To determine cold tolerance of *Arabidopsis*, 30-day-old transgenic seedlings and WT plants were subjected to low-temperature stress. Under normal growth, the characterization of transgenic seedlings was not significantly different from that of WT (Fig. 5B). The EL and MDA content of WT plant were significantly higher than the transgenic lines (Fig. 5C, D), indicating that WT plants suffered more severe cold damage than transgenic plants after low-temperature treatment. The above data indicated that overexpression of the *PavbHLH28* gene enhances the cold tolerance of *Arabidopsis*.

### Expression of cold-responsive genes in transgenic *Arabidopsis*

**Fig. 6:**
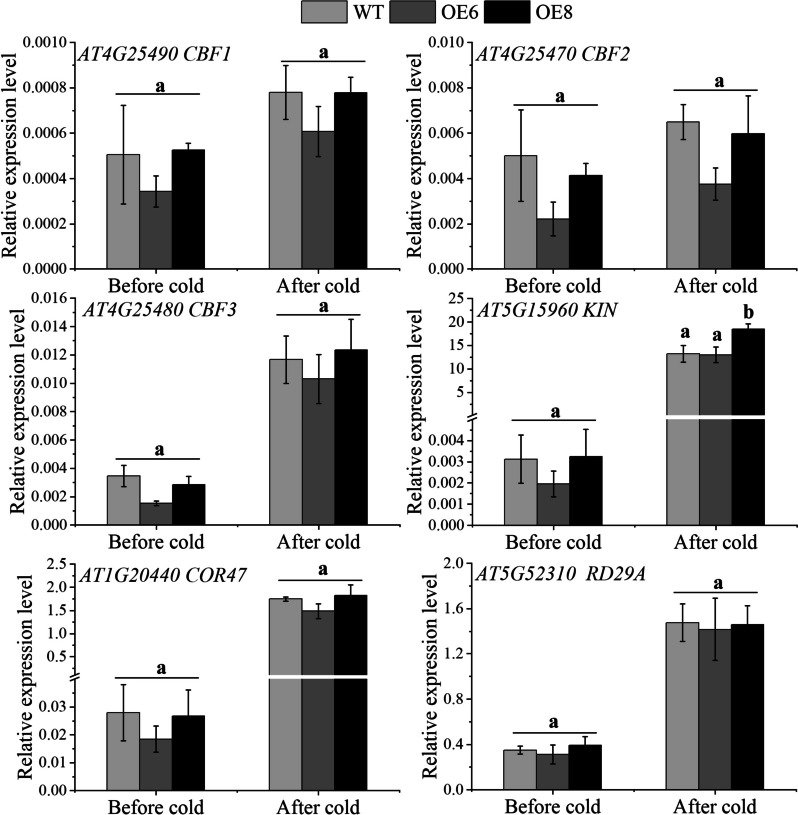
Analysis of stress-responsive genes in transgenic and WT plants under normal and low-temperature stress. WT stands for wild type plant. The OE6 and OE8 were *PavbHLH28* transgenic lines. Before cold and after cold represent before and after 4 °C treatment, respectively. Different letters indicate the significant differences of three replicates as determined by SPSS software (*p* < 0.05)

To further explore the mechanism of enhanced cold resistance of *PavbHLH28* over-expression lines, the expression levels of several cold stress-related genes in the *CBF-*dependent pathway of *Arabidopsis* were detected. Our results showed that cold stress-related genes were up-regulated in WT and transgenic plants under low-temperature treatment (Fig. 6). The *CBF3* gene was up-regulated 3.37, 6.71, and 4.35 times in WT, OE6, and OE8, respectively. The *KIN* gene has the largest up-regulated expression fold after low-temperature treatment. There was no significant difference in the expression of genes (*AtCBF1/2/3*, *AtKIN*, *AtCOR47*, *AtRD29A*) between WT and transgenic lines under low-temperature stress. The results revealed that *PavbHLH28* enhanced cold stress resistance in transgenic plants not by regulating CBF-related gene expression.

### The activities and expression levels of antioxidant enzyme in transgenic plants

**Fig. 7:**
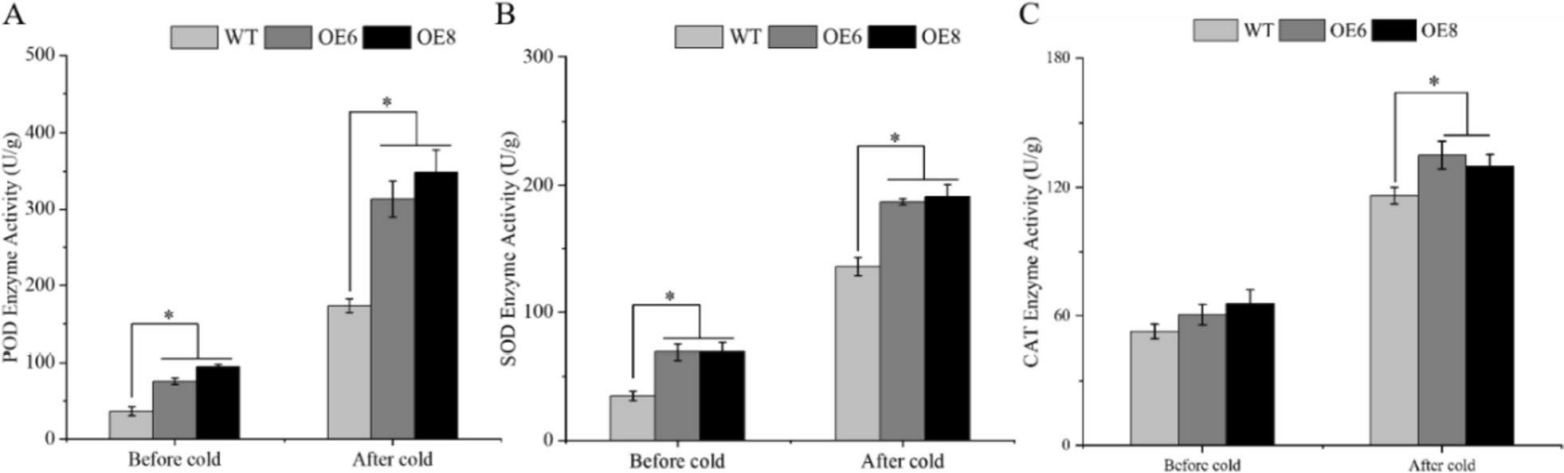
Activities of antioxidant enzymes in the transgenic lines and the WT after cold treatment. WT stands for wild type plant. The OE6 and OE8 were *PavbHLH28* transgenic lines. Before cold and after cold represent before and after 4 °C treatment, respectively. **A** and **B** represent SOD and POD enzyme activities, respectively

**Fig. 8:**
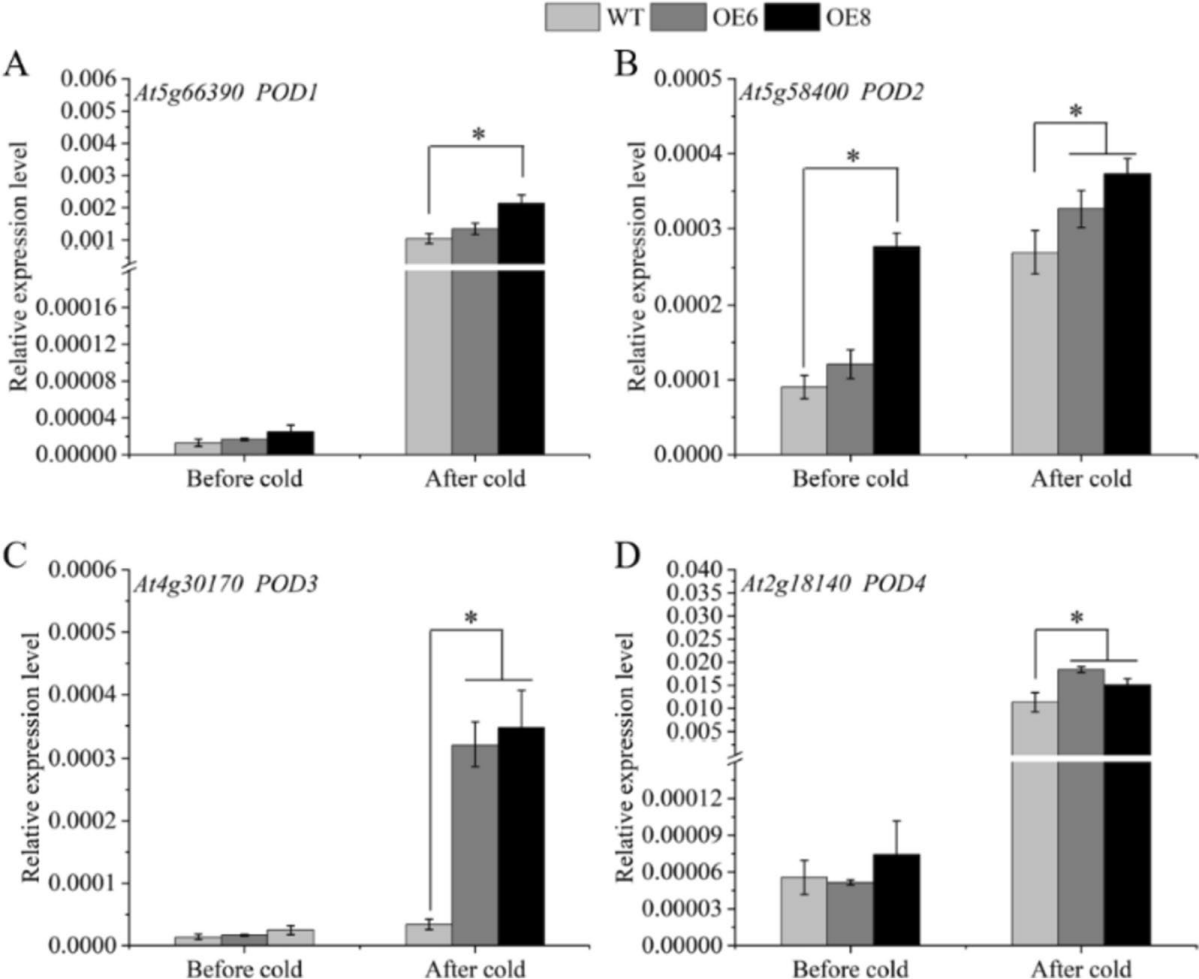
The gene expression analysis of *AtPOD* genes. WT stands for wild type plant. The OE6 and OE8 were *PavbHLH28* transgenic lines. Before cold and after cold represent before and after 4 °C treatment, respectively. * represents the significant differences of three replicates as determined by SPSS software (*p* < 0.05)

**Fig. 9:**
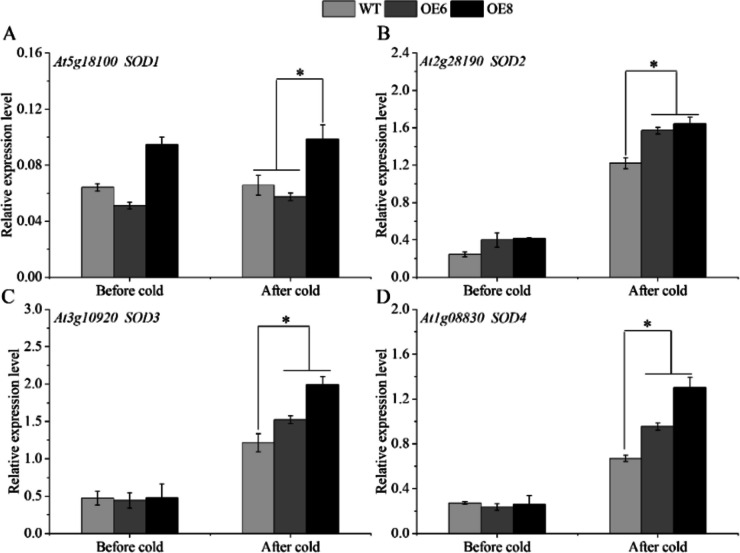
The gene expression analysis of *AtSOD* genes. WT stands for wild type plant. The OE6 and OE8 were *PavbHLH28* transgenic lines. Before cold and after cold represent before and after 4 °C treatment, respectively. * represents the significant differences of three replicates as determined by SPSS software (*p* < 0.05)

To explore whether transgenic strains enhance cold resistance by promoting the expression of antioxidant enzyme genes, the antioxidant enzyme activities of the transgenic lines and WT plants were measured after low-temperature treatment. The POD and SOD enzyme activities of the over-expression lines were significantly higher than those of the WT plants after cold stress (Fig. 7A, B). For CAT enzyme activity, there was no significant difference between transgenic strains and wild type before cold treatment, and CAT activity of transgenic strains was higher than that of WT plants after cold treatment (Fig. 7C). In addition, the expression levels of *POD* and *SOD* genes in plants before and after cold stress were detected. The highest transcription level is found in the *POD4* gene out of the four *POD* genes whose mRNA abundance was assessed. After the cold treatment, the *POD4* gene expression of the transgenic line was 1.78 times (OE6) and 1.47 times (OE8) that of the WT, respectively (Fig. 8D). Besides, the *POD3* gene expression had the largest fold change between transgenic lines and WT plants, the *POD3* gene expression of the transgenic line was 9.48 times (OE6) and 10.23 times (OE8) that of the WT, respectively (Fig. 8C). The expression of the transgenic line was much higher than that of the WT under cold treatment (Fig. 9), and for the *AtSOD* genes, all three (*AtSOD2/3/4*) showed significant increases in expression, except the *AtSOD1* gene.

### Transgenic plants accumulate less ROS

**Fig. 10:**
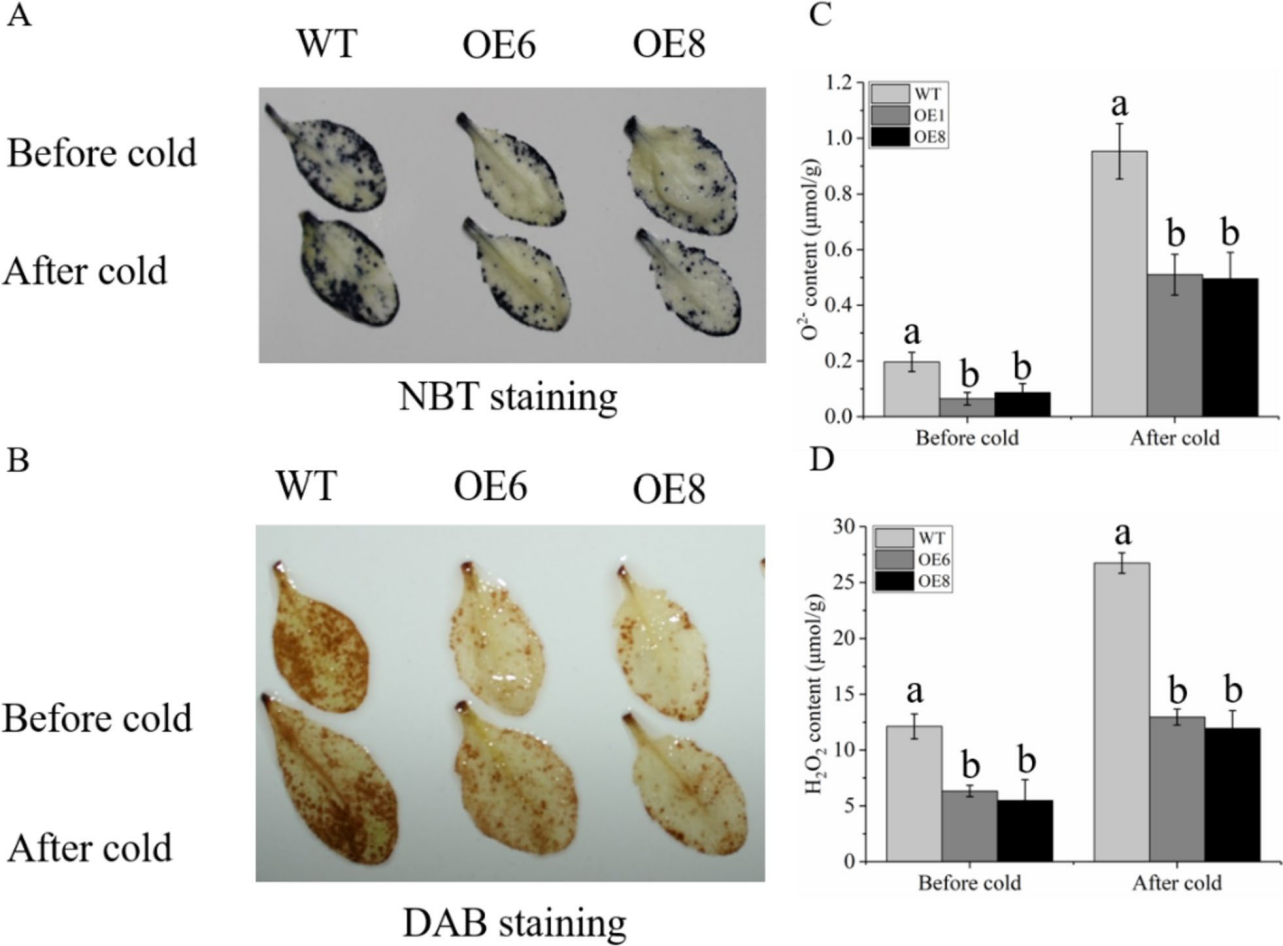
Analysis of ROS levels in WT and transgenic lines. WT stands for wild type plant. The OE6 and OE8 were *PavbHLH28* transgenic lines. **A**-**B** Histochemical staining with NBT (**A**) and DAB (**B**) for detecting the accumulation of O^2−^ and H_2_O_2_, respectively. **C** and **D** for the content of O^2−^ and H_2_O_2_ in the WT and transgenic lines. Before cold and after cold respectively represent before and after 4 °C treatment

Since the transgenic plants showed higher cold resistance than the WT, the NBT and DAB for histochemical staining were used to detect the accumulation of the two main ROS. The results showed that the WT strain had deeper staining (Fig. 10A, B). Further determination of the ROS content in the cells confirmed that it was consistent with the results of histochemical staining. The transgenic plants’ ROS content was significantly lower than that of the WT plants after a low-temperature treatment (Fig. 10C, D). This suggests that the transgenic plants’ high expression of antioxidant enzymes improved their capacity to scavenge ROS and prevented them from accumulating as much ROS.

### Analysis of expression levels related to proline metabolism genes and proline content

**Fig. 11:**
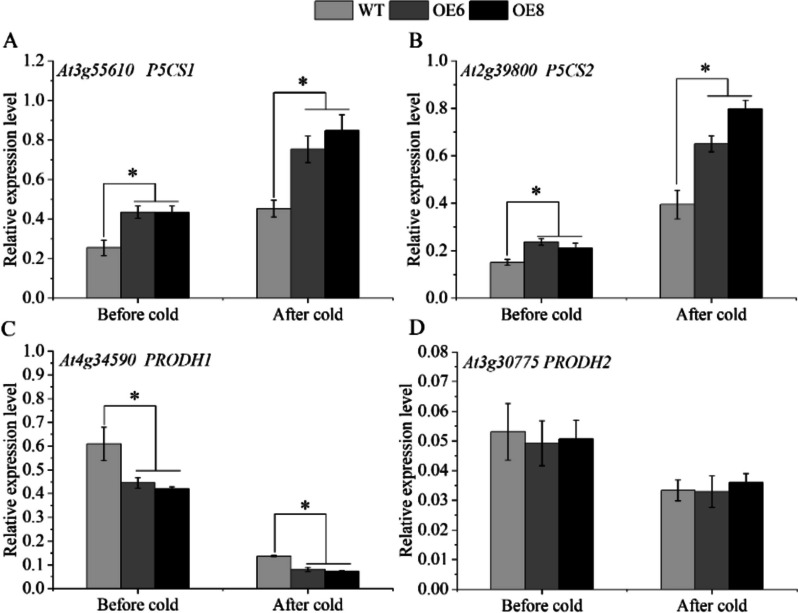
The expression analysis of proline synthesis and degradation genes. WT stands for wild type plant. The OE6 and OE8 were *PavbHLH28* transgenic lines. **A** and **B** represent the expression levels of proline synthase genes (*P5CS1* and *P5CS2*); **C** and **D** represent the expression levels of proline degradation genes (*PRODH1* and *PRODH2*); before cold and after cold respectively represent before and after 4 °C treatment; * represents the significant differences of three replicates as determined by SPSS software (*p* < 0.05)

**Fig. 12:**
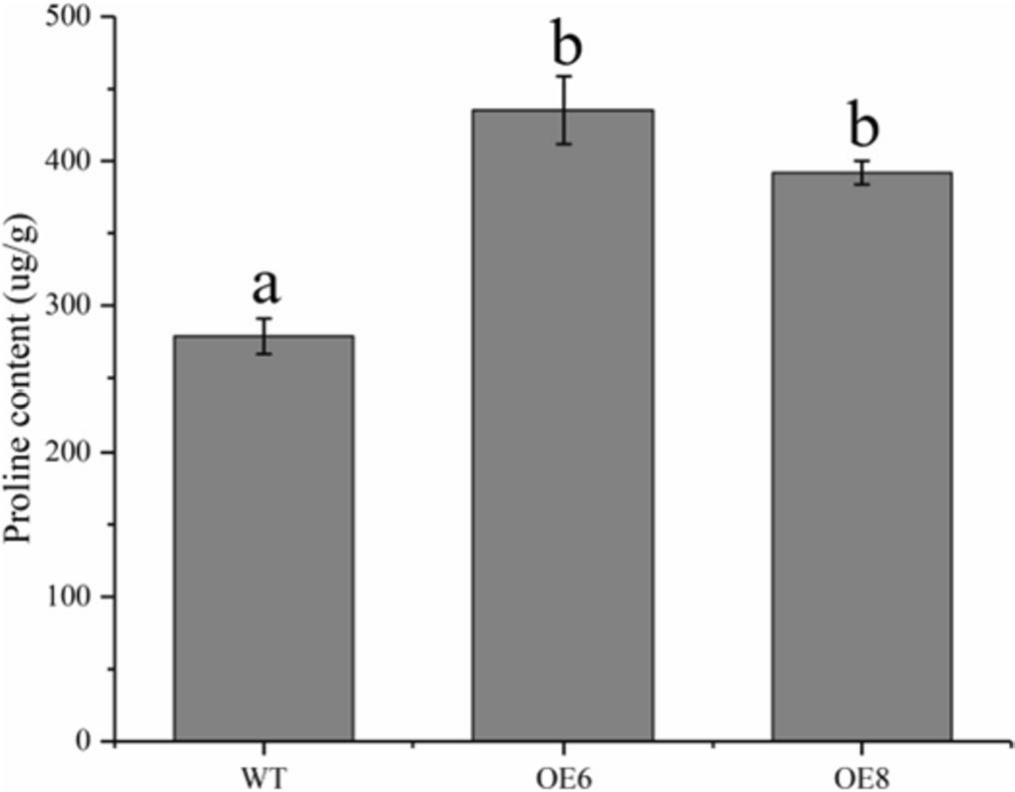
Detection of proline content in WT plants and transgenic lines after cold treatment. WT stands for wild type plant. The OE6 and OE8 were *PavbHLH28* transgenic lines. Different letters indicate the significant differences of three replicates as determined by SPSS software (*p* < 0.05)

To reveal the mechanism of cold resistance in transgenic lines, the expression levels of proline synthesis and metabolism genes in transgenic lines and WT were detected. The results showed that the expression abundance of proline synthetic genes in transgenic lines was significantly higher than that in WT plants with normal growth. After low-temperature treatment, the transcript level of proline synthase (*P5CS1/2*) increased rapidly, and the expression of *P5CS1* in the transgenic line was 1.66 and 1.87 times that of the WT plant, respectively (Fig. 11A). In addition, the gene abundance of the *P5CS2* in the transgenic line was 1.64 and 2.02 times that of the WT after low-temperature stress (Fig. 11B). In contrast, the proline dehydrogenase gene was significantly down-regulated. The *PRODH1* expression of the transgenic lines OE6 and OE8 were significantly down-regulated by 1.67 and 1.84 times compared with the WT plants under low-temperature stress (Fig. 11C). Meantime, compared with the WT plants, the transgenic line accumulated more proline after cold treatment (Fig. 12).

### PavbHLH28 promotes the expression of the *PavPOD2* gene

**Fig. 13:**
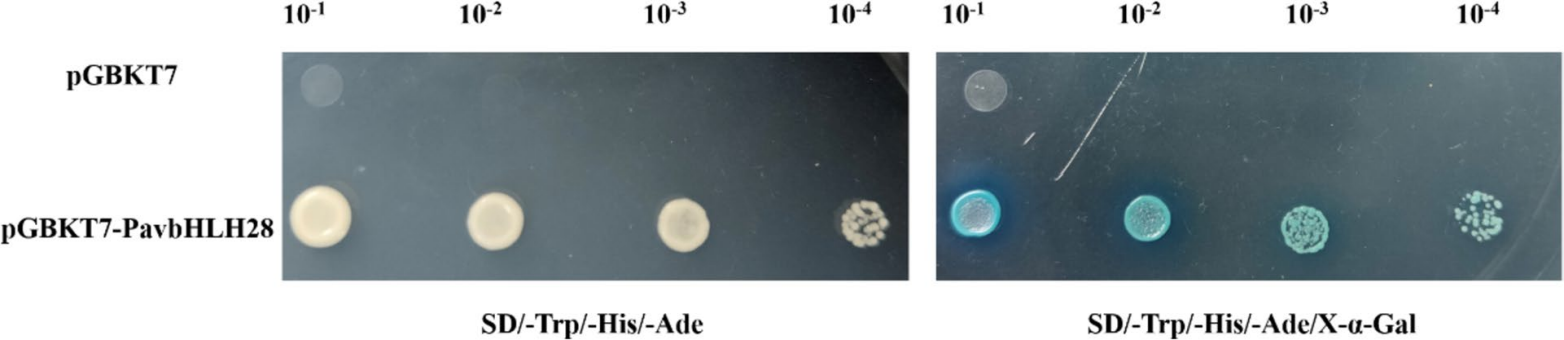
Validation of transcriptional activation of PavbHLH28 protein

**Fig. 14:**
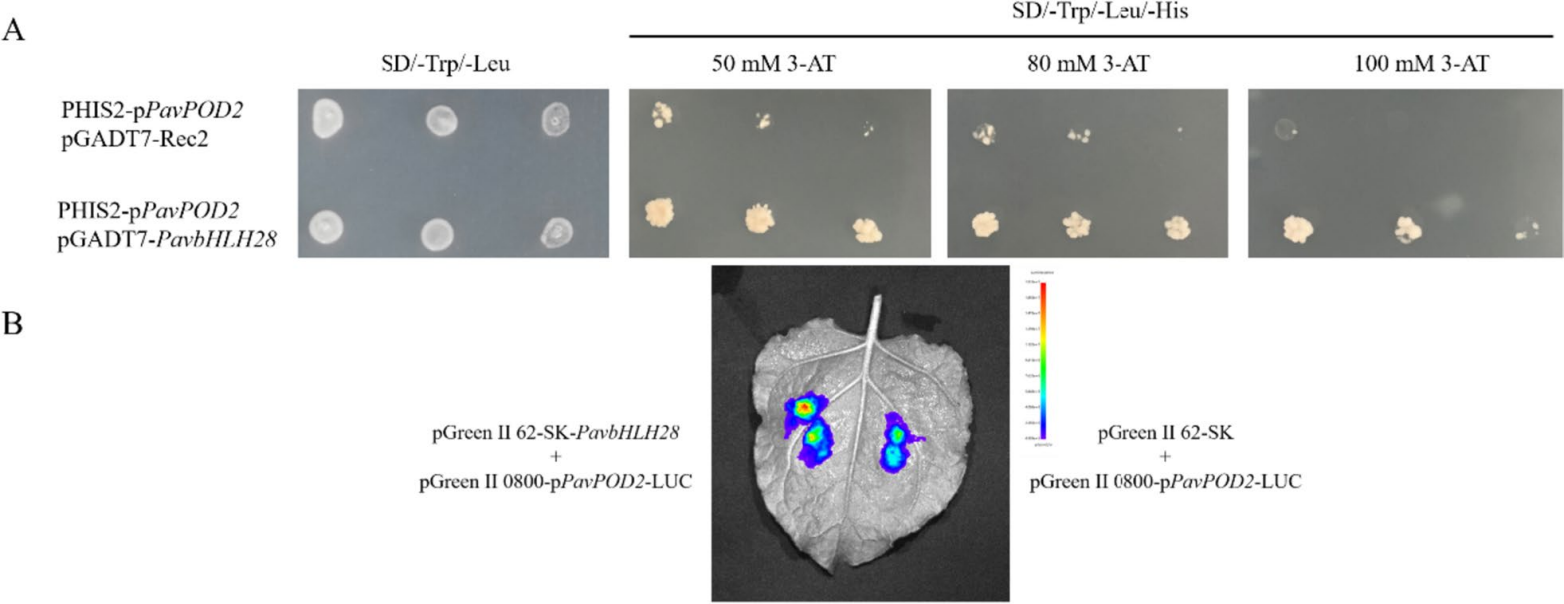
PavbHLH28 binds to the promoter of *PavPOD2.* **A** represents represents the result of yeast one-hybrid. **B** represents the result of dual luciferase assay

The transcriptional activity of the *PavbHLH28* indicated that its entire length possesses activation ability, which allowed us to confirm if the *PavbHLH28* directly regulated the expression of genes that encode antioxidant enzymes (Fig. 13). Subsequently, the *POD* gene promoter obtained by cloning was verified for interactions, and yeast one-hybrid and double-luciferase experiments confirmed that PavbHLH28 could directly bind the promoter of *PavPOD2* gene and promote its expression (Fig. 14).

## Discussion

Transcription factor (TF), also known as cis-acting factor, played a key role in the regulation of related physiological processes. It combined with the cis-acting element on the promoter to regulate the expression of downstream target genes [34]. The *bHLH* gene was involved in a variety of plants metabolic and physiological processes, such as growth and morphogenesis, hormonal signaling, and secondary metabolism [35]. In addition, the overexpression of *bHLHs* endowed plants with tolerance to salt, osmosis, freezing, cold and oxidative stress [27, 36]. The *ICE* gene was the MYC subfamily of the *bHLH* gene family. There have been many reports on the functions of plants involved in cold stress. Some recent research on *Rosa* [37], sweet potato [38], *Zoysia japonica* [39], *Saussurea involucrata* [40], *Malus domestica* [41], the homologous genes of *Arabidopsis ICE1* can enhance the cold resistance of plants. However, there are few studies on the involvement of *bHLH* gene in plant stress resistance in sweet cherry. The homology comparison of the *PavbHLH28* gene identified with *Arabidopsis* showed that it does not belong to the ICE transcription factor in this study. The study found that the expression of this gene could be highly induced by cold treatment, and the expression level was significantly increased under PEG and ABA treatment. It indicated that this gene may also play a role in the regulation of drought resistance in sweet cherry.

To reveal the function of *PavbHLH28* gene in cold tolerance, we genetically transformed *Arabidopsis thaliana* by constructing an overexpression vector. The transgenic lines showed higher cold tolerance following four days of 4 °C treatment, in accordance with the results. The electrical conductivity and MDA content were significantly lower than that of WT plants, indicating that the transgenic plants were less damaged by cold stress (Fig. 5). Thus, the results demonstrate that *PavbHLH28* has a positive role in cold stress. In most plants, the cold response was especially mediated by the *CBF*-*CORs* regulatory pathway, within which *CBFs* directly bound to the promoter of the *CORs* and enhanced their expression, thereby improved cold resistance [42]. To reveal the molecular mechanism of *PavbHLH28* enhancing cold tolerance of transgenic plants, the expression levels of cold stress-related genes were analyzed. Our results showed that the transcript levels of *CBF1-3* enhanced considerably when cold treatment, and therefore the upward trend of *CBF3* factor was the foremost vital (Fig. 6). However, there were no vital changes within the relative expression levels of *CBF1-3* genes between WT and transgenic plants. The other cold-responsive genes *COR47*, *RD29A* and *KIN* showed similar results with *CBFs* genes, only induced by cold treatment (Fig. 6). The above results demonstrated that *PavbHLH28* enhanced cold resistance by regulating the expression of *CBF*-independent pathway genes in transgenic *Arabidopsis*.

To reduce the over-generation of ROS under different abiotic stresses, plants developed a multifaceted antioxidant defense network. Among them, POD, SOD and CAT were essential for ROS detoxification, enabling plants to maintain better condition under abiotic stress [24, 43]. It was well known that many other bHLHs can bind to E-box elements, which retain the four nucleotides (ACGT or CANNTG) of the G-box core [44]. In addition, it was reported that *AtbHLH122* enhances tolerance to abiotic stress by inhibiting the expression of *CYP707A3* [45]. Many studies have also revealed that bHLH transcript factors directly regulated the expression of antioxidant enzymes and participated in the regulation of plant resistance. For example, *AtbHLH112* combined with E-box/G-box to control the expression of connected genes and increase the resistance of *Arabidopsis* to a range of abiotic stresses [27]. It was found that *Ah112* can directly bind to the *AhPOD* promoter and affect the drought resistance of plants in peanuts [46]. In citrus, *CsbHLH18* could directly activate *CsPOD* gene expression, but does not combine with *CsCAT* and *CsSOD* gene promoters [29]. In our research, we found that the activities of SOD and POD enzyme in transgenic plants was significantly higher than those of WT plants. Moreover, the gene expression analysis showed that the abundance of *AtPOD3/4* and *AtSOD2/3/4* genes in the transgenic plants was also higher than that of the WT after cold stress treatment (Figs. 8 and 9). This result also explained the lower ROS content in transgenic plants at low-temperature. The aforementioned finding suggested that the *PavbHLH28* gene may directly control the expression of antioxidant enzymes to boost transgenic *Arabidopsis’s* resistance to cold. Further experiments confirmed that *PavbHLH28* directly binds to the promoter of *PavPOD2* (Fig. 14), demonstrating that *PavbHLH28* could enhance plant cold resistance by promoting the expression of antioxidant enzyme.

Proline was an important plant penetrant that also acted as a ROS scavenger and stabilizer to protect macromolecules from denaturation [47]. It plays a key role in abiotic stress response including low temperature [48, 49]. The Δ-1-pyrroline-5-carboxylic acid synthase (*P5CS*) is the key enzyme that regulates proline biosynthesis [50]. In contrast, the *PORDH* gene mainly functions in proline catabolism [51]. In this study, the transgenic plants had a higher proline content after cold treatment (Fig. 12). Meanwhile, the transcription level of the proline synthase gene *P5CS1/2* in the overexpressed lines was higher than that in the WT plants. In addition, the expression abundance of the degrading enzyme *PORDH1* gene was lower than that of WT (Fig. 11). The results were also consistent with the higher proline content in transgenic plants. In summary, the *PavbHLH28* gene might increase the proline content by promoting the synthesis of proline and inhibiting proline degradation, thereby enhancing the cold resistance of transgenic plants.

## Conclusion

In the present research, *PavbHLH28* was highly induced to express under cold stress. Compared with WT plants, the transgenic plants of *PavbHLH28* showed a significant increase in proline content, accompanied by up-regulated expression of proline synthesis genes. The antioxidant enzyme activity and gene expression in transgenic plants were higher, which is consistent with the lower ROS content in *vivo*. The PavbHLH28 could enhance the expression of their genes by binding to the promoter of *PavPOD2*. In conclusion, The *PavbHLH28* could enhance cold tolerance in plants directly by increasing the activity of antioxidant enzymes, but not through the *ICE*-*CBF* pathway. Our research validated the function of the *PavbHLH28*, and provided new insights into the regulatory functions of the *bHLH* gene family members involved in resistance to cold stress in plants.

## Materials and methods

### Plant materials and treatments

The healthy adult sweet cherry (Brooks) trees growing under natural conditions were selected, and the healthy branches from the same part were collected for subsequent cold treatment and PEG treatment. Five branches were included in each biological replicate treatment sample. For cold treatment, the branches in a culture bottle containing water were placed in two artificial climate boxes. The photoperiod was set to 14 h of light/10 h of darkness, and the temperature was set to 4 and 25 °C, respectively. The leaves were collected 0, 1, 3, 6, 12, 24, 48, 72 h after treatment and stored at -80 °C. As a drought treatment, sweet cherry branches were soaked in 20% PEG 6000. The leaf tissues from the branches were collected at 0, 2, 4, 6, and 8 h after treatment. All processed leaf samples were immediately frozen in liquid nitrogen and stored at -80 °C. For phytohormones treatment, the branchs of healthy adult sweet cherry trees were selected and sweet cherry leaves were sprayed with 100 mg/L GA_3_, ABA, MeJA, and 10 mL per branch. The leaves were collected at 0, 1, 2, 3, 4, and 5 h after treatment, and then quickly frozen in liquid nitrogen and stored at -80 °C. For different tissue samples, the mature leaves, annual stems, blooming flowers, mature fruits, and mature fruit stalks were collected. The above experiments were all set with three biological replicates.

### Isolation and bioinformatics analysis of *PavbHLH28*

Total RNA was extracted from sweet cherry leaves according to the RNA extraction kit method (SENO, Zhangjiakou, China), and the cDNA was synthesized by PrimeScript™ RT reagent Kit with gDNA Eraser (TaKaRa, Dalian, China). According to the gene sequence in the sweet cherry genome (https://www.rosaceae.org/species/prunus/all), primers were designed to clone the ORF full-length sequence of *PavbHLH28*. The primers used are in Table S1. Use the online website (https://web.expasy.org/protparam/) to analyze the isoelectric point (PI), molecular weight (MW) of the protein. The evolutionary tree construction using plugins for Tbtools, including MUSCLE Wrapper, trimAL Wrapper and IQ-Tree Wrapper [52]. The sequences of bHLH genes were obtained from the iTAK online database, including *Arabidopsis thaliana*, *Oryza sativa* and *Prunus persica* (http://itak.feilab.net/cgi-bin/itak/index.cgi) [53].

### Expression profile analysis of *PavbHLH28*

Extracted the RNA from samples of different tissues and different treatments, and then first-strand cDNA was synthesized by PrimeScript™ RT reagent Kit with gDNA Eraser (TaKaRa, Dalian, China). PCR amplification was conducted in a volume of 10 µL, including 5 µL 2× PowerUp™ SYBR Green Master Mix (Thermo Fisher Scientific, USA), 0.5 µL of each forward primer (10 µM) and reverse primer (10 µM), 2 µL cDNA and 2 µL ddH_2_O. They were performed on the CFX Connect™ Real-Time PCR Detection System (Bio-Rad Laboratories, CA, USA). All experiments were performed in three biological and technical replicates. The elongation factor (EF) was used as the internal gene [54]. The relative expression levels of genes were calculated using the 2^−∆Ct^ method [55]. The primers are listed in Table S1.

### Subcellular localization analysis

The coding sequence of *PavbHLH28* with the stop codon removed was cloned into the pBWA(V)-Glosgfp vector to construct the pBWA(V)-*PavbHLH28*-Glosgfp GPF-fusion vector. The detailed preparation and transforming methods of protoplasts are completed by referring to the manual [56]. After transforming the *Arabidopsis* protoplasts, the fluorescence was observed using laser scanning confocal microscopy (Wuhan BIORUN BIO, Wuhan, China). The nuclear localization marker uses an NLS signal (protein sequence: MDPKKKRKV) vector [57].

### Transformation and characterization of transgenic plants

The ORF of *PavbHLH28* was inserted into the plant expression vector pBWA(V)KS-35s-GUS. It was transformed into GV3101 agrobacterium by freeze-thaw method, and pBWA(V)KS-35s-*PavbHLH28*-GUS was transformed into ecotype *Arabidopsis thaliana* Columbia (the plant specimen was deposited in the New York Botanical Garden, Barcode 1365355) by the inflorescence method. The T_0_ generation seeds were screened for Kana resistance, and qRT-PCR was used to detect gene expression. The homozygous transgenic plants of *Arabidopsis* T3 generation were used in this study. After extracting RNA from transgenic plants and synthesizing the first strand cDNA, qRT-PCR was used to detect gene expression in transgenic lines.The PCR amplification was conducted in a volume of 10 µL, including 5 µL 2× PowerUp™ SYBR Green Master Mix (Thermo Fisher Scientific, USA), 0.5 µL of each forward primer (10 µM) and reverse primer (10 µM), 2 µL cDNA and 2 µL ddH_2_O. They were performed on the CFX Connect™ Real-Time PCR Detection System (Bio-Rad Laboratories, CA, USA). The *actin* gene of *Arabidopsis* was used as an internal reference, and the relative expression of the *PavbHLH28* was calculated using 2^−∆CT^ [55].

### Analysis of cold-responsive and antioxidant enzymes genes

The transgenic lines and WT seedlings transplanted for 20 days were used in the cold treatment experiment. The gene expression of transgenic lines and WT plants was measured at 0 h (before cold) and 96 h (After cold) after 4 °C treatment. Total RNA of transgenic and WT plants before and after treatment was extracted. The method of total RNA extraction was referred to as the RNA extraction kit method (SENO, Zhangjiakou, China). Then, the first-strand cDNA was synthesized by PrimeScript™ RT reagent Kit with gDNA Eraser (TaKaRa, Dalian, China). The cold-responsive genes were selected to detect the expression levels of WT plants and transgenic lines, including *AtCBF1/2/3*, *AtCOR47 (*cold-regulated genes*)*, *AtRD29A* (RESPONSIVE TO DESICCATION 29A), *AtKIN* (kinesin) of the CBF-dependent pathway. The antioxidant enzyme genes referred to in the article, the *AtSOD1/2/3/4*, *AtPOD1/2/3/4* as candidate genes were detected in the expression levels [27]. The qRT-PCR amplification was conducted in a volume of 10 µL, including 5 µL 2× PowerUp™ SYBR Green Master Mix (Thermo Fisher Scientific, USA), 0.5 µL of each forward primer (10 µM) and reverse primer (10 µM), 2 µL cDNA and 2 µL ddH_2_O. They were performed on the CFX Connect™ Real-Time PCR Detection System (Bio-Rad Laborato-ries, CA, USA). All experiments were performed in three biological and technical replicates. The *actin* gene of *Arabidopsis* was used as an internal reference, and the relative expression of the gene was calculated using 2^−∆CT^ [55].

### Physiological analyses and histochemical staining

The MDA (Kit No. BC0020, Beijing Solarbio, China) and proline (Kit No. BC0290, Beijing Solarbio, China) content of transgenic and WT plants were determined at 96 h after 4 °C treatment. Each sample includes three biological replicates. Referring to the method published by Geng, the accumulation of H_2_O_2_ and O^2–^ was examined by nitroblue tetrazolium (NBT) and 3,3-diaminobenzidine (DAB) staining in situ [29]. The peroxidase (SOD), superoxide dismutase (POD) and catalase (CAT) activities of transgenic and WT plants were measured at 0 h (“Before cold”) and 96 h (“After cold”) after 4 °C treatment. The measurement methods of the above parameters referred to the kit protocol (Kit No. BC0020 for MDA, Kit No. BC0290 for proline, Kit No. BC0090 for POD, Kit No. BC0170 for SOD, Beijing Solarbio, China).

### Transcriptional activity analysis

To verify the activation activity of the *PavbHLH28*, the recombinant plasmid pGBKT7-*PavbHLH28* was constructed by inserting the full length of the *PavbHLH28* gene into the pGBKT7 vector. The recombinant plasmid and the negative control plasmid (pGBKT7) were transfected into AH109 yeast cells, respectively. Subsequently, the yeast colonies were inoculated on SD/-Trp/-His-/-Ade medium without or with 4 mg/mL X-α-gal, respectively, and incubated at 30 ℃ for 3 d. The yeast growth status was observed to determine the transcription activity. The primers used are in Table S1.

### Yeast one-hybrid and dual luciferase assay

Based on the sweet cherry genome, 2000 bp upstream of the *PavPOD2* gene was obtained as its promoter. The promoter of *PavPOD2* was cloned from sweet cherry leaf DNA and inserted into the pHIS2 vector. The full-length open reading frame of *PavbHLH28* was inserted into the pGADT7-Rec2 vector. The steps of yeast single hybridization experiments were referred to Matchmaker™ One-Hybrid Library Construction & Screening kit protocol (TaRaKa, Dalian, China). The *PavbHLH28* and promoter of *PavPOD2* gene were inserted into pGreen II 62-SK and pGreen II 0800-LUC vectors, respectively. Then, the recombinant plasmids pGreenII 62-SK-*PavbHLH28* and pGreen II 0800-p*PavPOD2*-LUC were constructed, and heat-stimulated transformation of *Agrobacterium 3101* was carried out to obtain the positive monoclones. The fluorescein luminescence intensity was detected using plant live imaging PlantView100 (BLT,Guangzhou, China) concerning the borrowed tobacco leaf transient transformation method [58]. The primers are listed in Table S1.

### Statistical analysis

The data were analyzed for significant differences using a student’s t-test at (*P* < 0.05) with SPSS 21.0 software (IBM, Chicago, USA). The graphs were constructed using Origin 9.0 (Origin Lab, Northampton, USA).

### Supplementary Information

**Additional file 1: Table S1.** The primers used in the study.

## Data Availability

All data generated or analysed during this study are included in this article and supplementary information files.

## Abbreviations

bHLH: Basic helix-loop-helix
EL: Electrolyte leakage
MDA: Malondialdehyde
ROS: Reactive oxygen species
CBF: C-repeat binding factor
ICE: Inducer of CBF Expression
CORs: Cold-Regulated genes
ORF: Open reading frame
TF: Transcription factor
