# Supplementary material for: Overexpression of PavbHLH28 from Prunus avium enhances tolerance to cold stress in transgenic Arabidopsis
Source: BMC Plant Biol. 2023 Dec 18;23:652. doi: 10.1186/s12870-023-04666-1 (PMC10726552; doi:10.1186/s12870-023-04666-1)
Supplement: Supplementary file 1 — Additional file 1: Table S1. The primers used in the study. [file 12870_2023_4666_MOESM1_ESM.docx]

| **Table S1 Primers used in this study** | |
| --- | --- |
| **Primer name** | **Primer sequence (5'-3')** |
| PavbHLH28-clone-F | ATGGCGGAGGAGTTTCAGG |
| PavbHLH28-clone-R | TCACCTGAAAGTTGCTCCAAATG |
| PavbHLH28-qF | TCGTCGTCAACACCCTCAGAT |
| PavbHLH28-qR | AAGTTATGGGAAAGCCAGAAGAA |
| PavbHLH28-GFP-F | cagtCGTCTCacaacatggcggaggagtttcaggc |
| PavbHLH28-GFP-R | cagtCGTCTCatacacctgaaagttgctccaaatg |
| PavEF-F | ATCCAGAGTAGCAGAACCAATCAC |
| PavEF-R | GTTAGGCATCCAGTCCCAGAAT |
| AtCBF1-F | GCAGCCAGCCAACCACA |
| AtCBF1-R | AGCGAAGCAATCCCACG |
| AtCBF2-F | AAGAAACCAGCGGGAAGGA |
| AtCBF2-R | CAAGCCGAGTCAGCGAAAT |
| AtCBF3-F | TCCTCAGGCGGTGATTATATTC |
| AtCBF3-R | TCCTTGTTTTCTTGTTTGGTTCT |
| AtRD29A-F | GCTTTCTGGAACAGAGGATGTA |
| AtRD29A-R | CGACTCTTCCTCCAACGTTATC |
| AtKIN1-F | TGGAGCTGGAGCACAACA |
| AtKIN1-R | GACCCGAATCGCTACTTGTTC |
| AtCOR47-F | TATCATGCCAAGACCACTGAA |
| AtCOR47-R | CAACGAAAGCCACAATAACAA |
| Atactin-F | GCACCCTGTTCTTCTTACCGA |
| Atactin-R | AGTAAGGTCACGTCCAGCAAGG |
| AtPOD1-F (At5g66390) | CTCACTAAGTTCAAGCGTC |
| AtPOD1-R (At5g66390) | GAATAGGGTCTGGTCACC |
| AtPOD2-F (At5g58400) | GGCAAGCCAGGTGCGTCAC |
| AtPOD2-R (At5g58400) | CCGGCTGTAGGATACGAC |
| AtPOD3-F (At4g30170) | AGCCGTCACGGCCTCTCTC |
| AtPOD3-R (At4g30170) | CAAGATTTGATCTGACGT |
| AtPOD4-F (At2g18140) | TCCGGGAGCCACACCATTGG |
| AtPOD4-R (At2g18140) | TGGTCGGAATTCAACAG |
| AthSOD1-F (At5g18100) | AACGAGGAAGAGCGTCATG |
| AthSOD1-R (At5g18100) | GCGTTTCCAGTTGATTTG |
| AthSOD2-F (At2g28190) | ATGTATCTCAACAGGACCAC |
| AthSOD2-R (At2g28190) | AGTGGTCAGACTAAGCTC |
| AthSOD3-F (At3g10920) | AGTGAAGGTGGTGGAGAGC |
| AthSOD3-R (At3g10920) | CATCTATACCCACCAGAG |
| AthSOD4-F (At1g08830) | ATGTCTACTGGTCCACATTTC |
| AthSOD4-R (At1g08830) | ATGGCCTCCCTTTCCGAG |
| P5CS1-F (At1g55610) | ATGATCTTATTTATGTTCTGC |
| P5CS1-R (At1g55610) | CACTATCTTCCGTCACTAT |
| P5CS2-F (At2g39800) | ACCAGAAGCACGGTCATTC |
| P5CS2-R (At2g39800) | CCATCTGAGAATCTTGTG |
| PRODH1-F (At4g34590) | AAGTGTCAGCATCACAAC |
| PRODH1-R (At4g34590) | CACGAAGAAATCATCAC |
| PRODH2-F (At3g30775) | GTATGACATTCCTGATGGAG |
| PRODH2-R (At3g30775) | GAAGGACAATGCATCTG |
| pPavPOD2-clone-F | GTCCTATTATTAGGAGGAGTGATCC |
| pPavPOD2-clone-R | GCTGCTTTCACTAAATCTAAAAGCTCC |
| PavbHLH28-62SK-F | tggcggccgctctagaATGGCGGAGGAGTTTCAGG |
| PavbHLH28-62SK-R | ctgcagcccgggggatccTCACCTGAAAGTTGCTCCAAATG |
| pPavPOD2-LUC-F | gcttgatatcgaattcctgcagGTCCTATTATTAGGAGGAGTGATCC |
| pPavPOD2-LUC-R | tctagaactagtggatccGCTGCTTTCACTAAATCTAAAAGCTCC |
| pPavPOD2-HIS-F | ctcactatagggcgaattcGTCCTATTATTAGGAGGAGTGATCC |
| pPavPOD2-HIS-R | tcgattcgcgaacgcgtgagctcGCTGCTTTCACTAAATCTAAAAGCTCC |
| PavbHLH28-AD-F | cattatggcccgggATGGCGGAGGAGTTTCAGG |
| PavbHLH28-AD-R | catgttttttcccgggTCACCTGAAAGTTGCTCCAAATG |
